# Breast Cancer Diagnosis Based on IoT and Deep Transfer Learning Enabled by Fog Computing

**DOI:** 10.3390/diagnostics13132191

**Published:** 2023-06-27

**Authors:** Abhilash Pati, Manoranjan Parhi, Binod Kumar Pattanayak, Debabrata Singh, Vijendra Singh, Seifedine Kadry, Yunyoung Nam, Byeong-Gwon Kang

**Affiliations:** 1 Department of Computer Science and Engineering, Faculty of Engineering and Technology (ITER), Siksha ‘O’ Anusandhan (Deemed to be University), Bhubaneswar 751030, India; binodpattanayak@soa.ac.in; 2 Centre for Data Sciences, Faculty of Engineering and Technology (ITER), Siksha ‘O’ Anusandhan (Deemed to be University), Bhubaneswar 751030, India; manoranjanparhi@soa.ac.in; 3 Department of Computer Applications, Faculty of Engineering and Technology (ITER), Siksha ‘O’ Anusandhan (Deemed to be University), Bhubaneswar 751030, India; debabratasingh@soa.ac.in; 4 School of Computer Science, University of Petroleum and Energy Studies, Dehradun 248007, India; vijendra.singh@ddn.upes.ac.in; 5 Department of Applied Data Science, Noroff University College, 4612 Kristiansand, Norway; skadry@gmail.com; 6 Artificial Intelligence Research Center (AIRC), Ajman University, Ajman 346, United Arab Emirates; 7 Department of Electrical and Computer Engineering, Lebanese American University, Byblos P.O. Box 13-5053, Lebanon; 8 MEU Research Unit, Middle East University, Amman 11831, Jordan; 9 Department of ICT Convergence, Soonchunhyang University, Asan 31538, Republic of Korea

**Keywords:** breast cancer diagnosis, Fog computing, IoT, convolutional neural network (CNN), deep transfer learning (DTL)

## Abstract

Across all countries, both developing and developed, women face the greatest risk of breast cancer. Patients who have their breast cancer diagnosed and staged early have a better chance of receiving treatment before the disease spreads. The automatic analysis and classification of medical images are made possible by today’s technology, allowing for quicker and more accurate data processing. The Internet of Things (IoT) is now crucial for the early and remote diagnosis of chronic diseases. In this study, mammography images from the publicly available online repository The Cancer Imaging Archive (TCIA) were used to train a deep transfer learning (DTL) model for an autonomous breast cancer diagnostic system. The data were pre-processed before being fed into the model. A popular deep learning (DL) technique, i.e., convolutional neural networks (CNNs), was combined with transfer learning (TL) techniques such as ResNet50, InceptionV3, AlexNet, VGG16, and VGG19 to boost prediction accuracy along with a support vector machine (SVM) classifier. Extensive simulations were analyzed by employing a variety of performances and network metrics to demonstrate the viability of the proposed paradigm. Outperforming some current works based on mammogram images, the experimental accuracy, precision, sensitivity, specificity, and f1-scores reached 97.99%, 99.51%, 98.43%, 80.08%, and 98.97%, respectively, on the huge dataset of mammography images categorized as benign and malignant, respectively. Incorporating Fog computing technologies, this model safeguards the privacy and security of patient data, reduces the load on centralized servers, and increases the output.

## 1. Introduction

Breast cancer is the most frequent form of cancer in women and is responsible for the deaths of approximately 36% of all women annually. Among both sexes, breast cancer has the second-highest incidence and fatality rates [1,2,3]. According to the World Health Organization (WHO), breast cancer is the second leading cause of death in women globally. The best way to save lives and cut medical costs is through the early identification of breast cancer. The technology used to detect and diagnose breast cancer is constantly advancing, giving patients better and less intrusive alternatives. Mammography is the single most important factor in reducing deaths from breast cancer [4,5,6].

**Figure 1:**
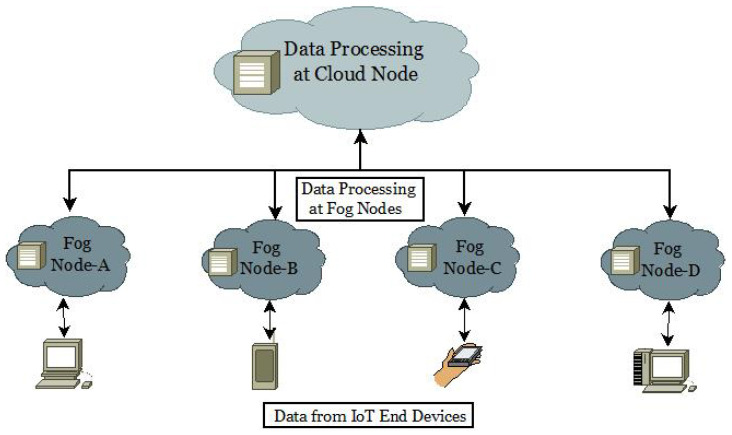
Cloud-based Fog distributions to IoT end devices.

As smart medical devices continue to advance rapidly, the Internet of Things (IoT) has many potential uses in the healthcare industry. The present approach is based on centralized communication with Cloud-based servers. However, this architecture exacerbates existing risks to privacy and security. Fog computing is a form of Cloud computing that moves data, processing, computation, and applications from the Cloud to the periphery of a network [7,8]. Instead of relying on a centralized server farm, “Fog computing” deploys its applications throughout a network. CISCO created Fog computing to incorporate Cloud computing into the network and accommodate previously unsupported file types. Moving host nodes, changing data centers, exchanging information, and ensuring that data are secure and reliable are all issues arising from Fog computing. In addition, the aged, the chronically ill, and the physically disabled increasingly demand a healthcare system that can provide a reliable, all-encompassing continuous health monitoring system [9,10]. Several academic research articles claim that remote health monitoring systems finally allow doctors to keep tabs on their patients in a timely and accurate manner [11,12,13]. In Figure 1, the diffusion of Cloud-based Fog to IoT end devices is shown.

Breast cancer diagnosis and prognosis research publications are available in print. Despite their prevalence, mammography images have not been the focus of extensive studies on the classification of breast cancer. Ensemble deep learning (EDL) models are flexible, but they still need image pre-processing and segmentation techniques for breast cancer classification. In this research, deep transfer learning (DTL) was used in The Cancer Imaging Archive (TCIA) repository to create a unique remote automated assistance system for identifying and categorizing breast cancer. The data were preprocessed before being introduced to the model. In order to boost prediction accuracy, prominent deep learning (DL) techniques such as convolutional neural networks (CNNs) were combined with transfer learning (TL) techniques such as ResNet50, InceptionV3, AlexNet, VGG16, and VGG19. Then, the support vector machine (SVM) classifier was added to classify into binary categories. The proposed method was put through rigorous simulation testing to establish its viability. Researchers are also interested in learning how IoT and Fog computing might be utilized to maintain individual patients’ information, lighten the load on centralized systems, and boost productivity.

The following is a list of this work’s main contributions:

- Fog computing along with Cloud computing and IoT for real-time analysis and IoT monitoring system installation;
- An automatic, remote diagnosis of benign and malignant breast cancer in different people;
- A model for real-time breast cancer diagnosis using DTL was trained using images from mammograms;
- The predictive and network analysis performance of the proposed system is shown and analyzed;
- Predictive analytics by modeling and simulating IoT–Fog–Cloud environments;
- Introducing the findings and comparing them with prior research to emphasize the unique contribution of the current study.

The remaining text is organized in the manner listed below. This document’s Section 2 provides details on the relevant work. Section 3 gives the approaches used in this proposed work in depth. The suggested work’s intricate architectural design and the implementation of the experimental setup are covered in Section 4. A discussion of the findings and analyses is included in Section 5. We conclude the suggested methodology in Section 6 with recommendations for the future.

## 2. Literature Study

McKinney et al. [14] proposed an AI system for breast cancer prediction that outperforms human specialists. The authors curated a large representative dataset from the United Kingdom and a large enriched dataset from the United States to evaluate its performance in a clinical scenario. The authors showed that the system is transferable from the UK to the US. The area under the receiver operating characteristic curve (AUC-ROC) for the AI system was higher than that of the typical radiologist according to an independent study of six radiologists.

Alahe and Maniruzzaman [15] presented a completely automatic method for detecting breast cancer utilizing two well-known filters, the detail enhanced (DE) and Gaussian blur (GB) filter for preprocessing. Here, a CNN classifier was used to conduct classification. The findings show how well the proposed model performs when used in the Breast Histopathology Image dataset, a publicly accessible dataset.

Xu et al. [16] suggested an enhanced semi-supervised tumor detection technique based on fuzzy c-means clustering with 10 3-D and 2-D tumor characteristics. Fog computing distributes sophisticated data processing. First, the authors 3-D-modeled and segmented tumors using FRFCM. The troublesome 3-D and 2-D tumor shape features were modeled to create feature vectors. The scientists created an upgraded semi-supervised FCM clustering to aid tumor identification using landmark data from common databases and experts. The trials employed CT images of 143 people and 452 cancers.

Zhu et al. [17] employed DL to develop a method for improving the quality of low-dose mammography images. The fundamental goal of the CNN model used for low-dose mammography is noise reduction. With experience, low-dose mammography can provide a high-quality picture. The TCIA repository’s experimental data sets were utilized to verify this method. It will promote the use of modern deep learning techniques in low-dose mammography.

Chougrad et al. [18] introduced task collaboration using multi-label picture categorization. The authors also shared an innovative approach to TL adjustment. This method uses end-to-end image representation learning to adapt a pre-trained CNN to a fresh challenge. In addition, they suggested a label selection technique tailored to this issue that calculates the best possible degree of certainty for each visual thought. On the Mammographic Image Analysis Society Database (MIAS), INBreast, Breast Cancer Digital Repository (BCDR), and Curated Breast Imaging Subset of the Digital Database for Screening Mammography (CBIS-DDSM) benchmark datasets, they demonstrated the usefulness of their methodology and obtained results that were superior to those of other widely used baselines.

Allugunti [19] suggested a computer-aided diagnosis (CAD) approach for classifying patients into three categories (non-cancerous, no cancer, and cancer) and making a diagnosis using a database. The author studied and examined three efficient classifiers for the classification stage: CNN, SVM, and random forest (RF). A higher success rate in categorizing was made possible by the author’s investigation into the effects of the mammography images being pre-processed beforehand.

Goen and Singhal [20] employed the CNN approach to separate malignant and noncancerous breast cancer images. Deep CNNs (DCNNs) were used to eliminate descriptive features automatically. The project will speed up analysis by aiding in breast cancer diagnosis and categorization. Despite years of experience, specialists sometimes disagree with radiologists’ tumor detection from histological images. Computer-aided picture diagnosis will boost expert supervision consistency. Automatic and exact breast cancer picture taxonomy relies on histopathology tumor identification.

Canatalay et al. [21] used a different dataset to develop three standard approaches. The models used here used deep learning to identify and categorize breast cancer in X-rays. The proposed model may accurately identify benign or malignant X-ray mass regions. The proposed model was analyzed using open-source TCIA X-ray images. Data were preprocessed before entering the model. TL improves the prediction accuracy. The recommended model was tested using detailed simulations. The model has the highest ResNet-164 training and validation accuracy.

Pourasad et al. [22], based on ultrasound scans, developed a breast cancer detection system. Six techniques were utilized to segment ultrasonic images. Photographs were analyzed using fractals. Classification algorithms such as SVM, K-nearest neighbors (KNN), decision trees (DT), and naive Bayes (NB) were also used. The CNN uses ultrasound images to categorize breast cancer. The high-potential CNN algorithm used in this study can recognize breast cancer in ultrasound images. The tumor’s origin can be located using the second CNN model. The tumor’s location and size were determined using morphological operations. These findings can be applied to monitor patients and stop the spread of disease.

Kavitha et al. [23] established an optimal multi-level thresholding-based segmentation with a DL-enabled capsule network (OMLTS-DLCN) model for employing digital mammograms to diagnose breast cancer. Mammogram noise was reduced by adaptive fuzzy (AFF)-based median filtering in the OMLTS-DLCN model. Kapur’s optimal multilevel thresholding with shell game optimization (OKMT-SGO) was applied to breast cancer segmentation. The method employs a feature extractor based on CapsNet and a BPNN classification model to identify breast cancer. OMLTS-DLCN diagnostic results were examined using DDSM and Mini-MIAS as standards. The experimental results demonstrate that, on the DDSM and Mini-MIAS datasets, the OMLTS-DLCN model has superior accuracy.

Jabeen et al. [24] suggested a novel framework based on DL and best-chosen criteria for classifying breast cancer from ultrasound images. There were five main phases to the proposed procedure. The output layer of a pre-trained DarkNet-53 model was tweaked using the additional dataset’s classes. The new model was trained using TL and features from the global average pooling layer, with the best features chosen using two improved optimization techniques. Breast ultrasound images (BUSIs) were used in the experiment, and their greatest accuracy was 99.1 percent. In comparison to prior methods, the suggested framework performed admirably.

Jasti et al. [25] discussed an evolutionary machine learning (ML) and image processing method for categorizing and identifying breast cancer. This approach integrates image preprocessing, feature extraction, feature selection, and ML to identify skin conditions. The geometric mean improves the image quality. AlexNet pulls features. Relief picks features. The model employs machine learning techniques to categorize and detect diseases. The experiment used MIAS data. The suggested method used image analysis to diagnose breast cancer.

Qi et al. [26] developed an automated breast cancer diagnosis. The phone-based system diagnoses ultrasound photos. It has three subsystems. The first subsystem rebuilds high-quality images from low-noise shots and uses stacked autoencoders and generative adversarial networks (GANs). The second subsystem employs convolutional neural networks to recognize harmful images. The third subsystem lowers false negatives and model performance concerns. GANs differentiate false from authentic negative samples. The method used 18,225 breast ultrasound images and 2416 ultrasound reports. Our system performs like humans, according to experiments. Mobile breast cancer diagnostics are new. The online system assists with breast cancer screening, diagnosis, early treatment, and mortality.

Ragab et al. [27] produced an ensemble deep-learning-enabled clinical decision support system for breast cancer diagnosis and classification (EDLCDS-BCDC) that endorsed USI breast cancer screening. Wiener filtering and contrast enhancement are the first two steps in this approach. Image segmentation also uses Kapur’s entropy (KE) and the chaotic krill herd algorithm (CKHA). SqueezeNet, VGG-16, and VGG-19 were used for feature extraction. The multilayer perceptron (MLP) model using cat swarm optimization (CSO) identifies images based on the presence of breast cancer. Numerous simulations on benchmark databases show that the EDLCDS-BCDC strategy outperforms more current approaches.

## 3. Materials and Methods

This work aimed to develop and train a DTL-based mammography image model. We obtained and examined many cancer imaging data from the TCIA database to train the model. Various network and performance matrices were then used to assess the model’s performance. In addition, we describe the dataset and the methods that were employed in this proposed framework.

### 3.1. Dataset Description and Acquisition

**Figure 2:**
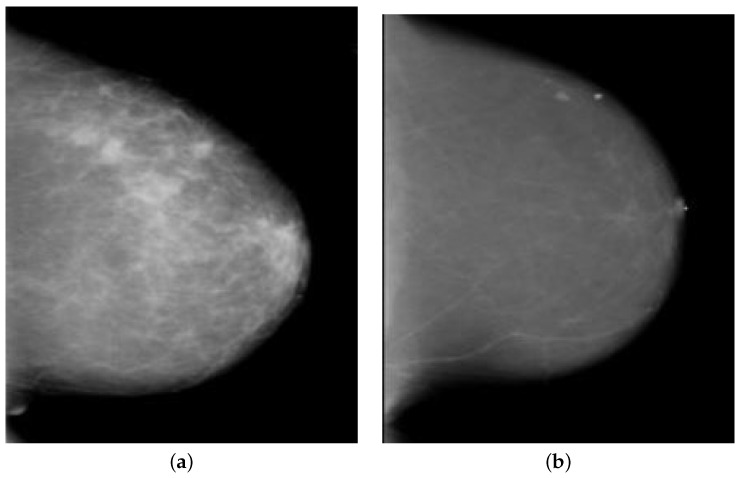
The sample breast mammography images taken from the DDSM dataset as an example. (**a**) The malignant image; (**b**) The benign image.

The TCIA open-source web database has a massive collection of medical images connected to cancer [28], including disease-specific sets of Digital Imaging and Communications in Medicine (DICOM) image files. The TCIA repository includes a number of datasets comprising mammography images, including CBIS-DDSM, VICTRE, CMMD, CDO-CESM, TCGA-BRCA, Breast Diagnosis, etc., totaling 6888 individuals and 570,579 images. Since the Digital Database for Screening Mammography (DDSM) is publicly available through TCIA for the purpose of diagnosing breast cancer, no informed permission or consent was required for its experiments [29]. Using supervised learning, it might learn from radiologists’ interpretations of pathology reports to determine whether or not a patient’s breast tissue is malignant, benign, or healthy. From a total of 3568 mammogram images (1828 malignant and 1740 benign), we considered data from 1784 random images (914 malignant and 870 benign), as indicated in Table 1 and Figure 2. As can be seen in Table 1, the dataset was split 70:10:20 across the training, validation, and testing phases. The 5-fold cross-validation method was used to evaluate the preprocessed dataset in this research.

The public has a very polarized view of breast cancer. When data are inconsistent, it is not uncommon to find missing or unimportant values, graphs, etc. Before modeling and analysis can occur, raw data must be cleaned, extracted, analyzed, and preprocessed. The primary goal of pre-processing mammography images is to enhance image quality by removing or significantly reducing the need to address unfavorable components of the image’s history. Mammograms are complex medical images that may be read and understood. As a result, preprocessing plays a crucial role in improving quality. The images were converted from the DICOM format used for the original mammograms to the PNG format while preserving the original pixel values using an automated preprocessing step. Using a computer vision-based technique, we converted the DICOM images to portable network graphics (PNGs) and extracted all the patient data into a CSV file. Mammograms for DDSM were expertly captured and archived in DICOM format. Only 3568 images are included in the DDSM dataset. The CNN with TL approaches was trained on the CBIS-DDSM dataset employing the same image preparation methods, including shearing, shifting, horizontal flipping, and scaling rotation, as those of Guan et al. [30] to enable the generalization of the classification of synthetic mammography images. CBIS-DDSM mammograms were acquired in high-quality DICOM format. In addition, we preprocessed images from the CBIS-DDSM dataset using the same technique as Guan et al. [30]. In ML, data augmentation refers to the process of adding modifications to an existing dataset in order to improve its diversity and quantity. Rotations, translations, flips, zooms, and other operations that change the images are all examples of transformations. As a result, we did not employ data augmentation.

### 3.2. Methodologies

DL is a method for learning how to map raw data to the desired output using several hierarchical neural networks. The use of CNN architecture requires the proposed DL framework [31]. To replicate breast cancer detection, layers of computer units from the DL model were added to them. A method for transferring information from one related topic to another is called TL, and five TL networks are present in the system: ResNet51, InceptionV3, AlexNet, VGG19, and VGG16. They are used if the data set is insufficient for training any network’s parameters. The five pre-trained CNNs that make up the proposed CNN architecture are described in this section.

The basic residual structure is combined with 50 deep layers in ResNet-50. It avoids degradation and features numerous convergence filters trained on millions of photos. It is the kind of DL architecture where the network gets deeper. This feature is distinct from the earlier models. The residual block, which feeds residual information to the following layers, is added to the model to generate ResNet-50. The ResNet-50 classic model is no longer applicable to this feature. ResNet-50, in summary, is formed from the term “residual network.” Residual blocks comprise the ResNet-50 Network, which was developed by including some shortcuts into the conventional network [32]. The value is taken as an input and placed through the convolution in the residual block. A function g(z) and an activation–convolution series are obtained. Then, the function g(z) is created by adding the original input value of *z* to the function:

(1) h(z)=g(z)+z

It should be noted that the function h(z) is equal to the function g(z) in the traditional convolution process. However, the original data are also included when the convolution technique is applied to the input of this network.

The network depth and width increase in InceptionV3 to increase computational capacity. It has been trained on 1 million photos and contains 48 layers with skipped connections. The starting modules are repeated using max pooling to reduce dimensionality. The architecture of InceptionV3 is divided into modules. Each module includes maximum pooling and convolution of varying sizes. Thus, “inception” is the name given to each module. The model, commonly known as GoogLeNet, has exactly 9 inception blocks. InceptionV3 is, in essence, a kind of CNN model [33]. There are numerous convolutions and maximum pooling steps in it. The task includes a fully connected neural network at the bottom layer. Maximum pooling is employed in the pooling layers, while ReLU is used as the activation function.

One of the most well-known CNN designs is AlexNet. Its structure consists of several layers [34]. It has five folded layers and three subordinate, maximum associating, and associating layers. First, AlexNet’s initial training is sent. The second step’s feature element is attached to a newly generated second-half section, forming the network’s basic design. In completely connected layers, 50% subtraction is used to deactivate the learning units erratically. As a result, SoftMax improves generalization performance by changing weights after each training iteration. The loss function is cross entropy. To minimize the loss, SoftMax is employed. It functions as a SoftMax classifier very well. The fact that it is employed in situations where more than two classifications are necessary is its most significant characteristic.

A structure resembling CNN is the visual geometry group (VGG). By sequentially substituting several tiny 2 × 2 filters at the max-pooling layer with 3 × 3 kernel-sized filters at the convolution layer, the system outperforms the AlexNet framework. The first convolutional layer in the kernel-sized filter is represented by 11 whereas the second convolutional layer is represented by 5. A sigmoid function activates the output, containing two FC layers overall. The well-known VGG models are the VGC16 and VGC19. The VGG19 model has 19 layers whereas the VGG-16 model only has 16. The key distinction between the two models is that each of the three convolutional blocks in the VGG19 model contains an additional layer [35,36]. ReLU is employed in both input and hidden layers scenarios, whereas sigmoid is used as an activation function in the output layers.

The activation function (AF) uses a weighted sum and bias to activate neurons. This function non-linearizes the neuron’s output and input, facilitating learning and advanced performance. The various AFs might take on a linear, ReLU, tanh, Leaky ReLU, SoftMax, sigmoid, or logistic function. The linear function’s equation resembles a straight line. Assuming that all layers are linear, the final activation function is a linear function of the first layer’s input. The sigmoid function outputs 0-1 and accepts any real value as input. The TanH, or the tangent hyperbolic function, performs better than the sigmoid function and mathematically adjusted sigmoid function. Both are similar and related. Most neural network (NN) hidden layers use the ReLU activation function. Functions produce nonlinear neural output. ReLU, a popular DL-AF, delivers great results. The diminishing gradient is fixed. Sigmoid DL-AF is frequent. Sigmoid transforms small and large integers into values near 0 and 1. Popular AFs include ReLU and sigmoid for binary classification. Leaky ReLU solves the dying ReLU problem. Softmax is a sigmoid function that helps with categorization [37,38]. Formulas for evaluating these functions are:

(2) Linear(z)=cz

(3) $$Sigmoid\left(z\right)=\frac{1}{1+e^{-z}}$$

(4) $$Tanh\left(z\right)=\frac{e^{z}-e^{-z}}{e^{z}+e^{-z}}=2*Sigmoid\left(2z\right)-1$$

(5) Relu(z)=max(0,z)

(6) LeakyRelu(z)=max(0.1∗z,z)

(7) $$SoftMax\left(z\right)=\frac{e^{z_{p}}}{\sum_{q}e^{z_{q}}}$$

where Linear(z), Sigmoid(z), Tanh(z), Relu(z), LeakyRelu(z), and SoftMax(z) are the AFs for linear, sigmoid, Tanh, ReLU, Leaky ReLU, and SoftMax, whereas max() finds the maximum value in between and *c* is any constant. This work used the sigmoid, ReLU, and softmax functions in various layers of different DTL approaches.

## 4. Proposed Work

**Figure 3:**
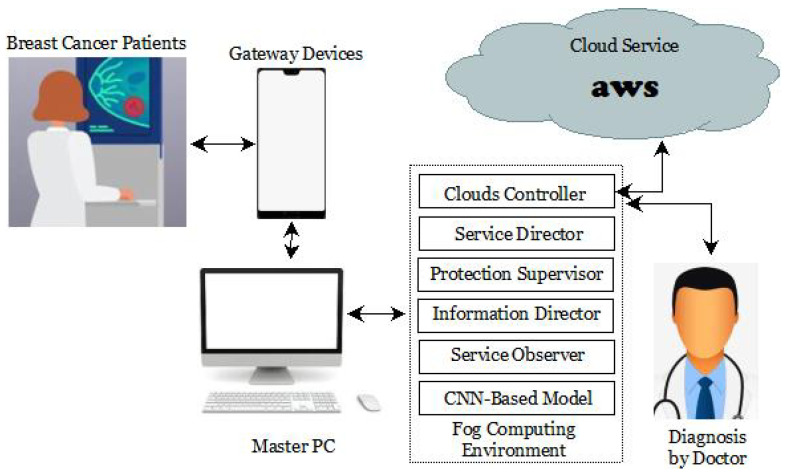
The proposed work architecture.

Detailed descriptions of the proposed work’s architecture, design, implementation, and operational method may be found here. This proposed work’s architecture comprises numerous components, each of which is discussed in more detail below and shown in Figure 3. The suggested study integrates IoT, Fog, and Cloud computing techniques for the finest predictive analytics.

### 4.1. Components Used

The IoT end devices, gateway devices, a master PC, Fog worker nodes, and Cloud data center nodes are all necessary hardware components for the proposed operation. Breast cancer patients’ data are detected by IoT end devices and transferred to gateway devices. The gateway device, i.e., a smartphone or computer to a tablet or laptop, accepts and transmits patient data to the system’s master or worker nodes. The gateway devices function similarly to Fog devices. The gateway devices provide job requests to the master PC, which either distributes the tasks to the available worker nodes via an information director or runs them through a trained CNN to generate output. When the master PC and Fog worker nodes reach their capacity, the master PC becomes a gateway device and redirects traffic to other Cloud-data-center nodes using a Cloud controller to handle the extra load. When a gateway device or master PC requests information, the data are processed by a Fog worker node, which then returns the results using the CNN-based model that it has been trained on. Raspberry Pi computers served as the Fog worker nodes in this investigation. Cloud resources are accessed via the Cloud-data-center node as and when required. The information director, service director, protection supervisor, Clouds controller, service observer, and a CNN-based model are only a few of the software components that are part of the proposed work. Data collected from discovered IoT devices will be analyzed by the information director. In addition, it has the flexibility to adjust the rate at which data are transferred and combine information from many sources. The information director is responsible for determining which Fog worker nodes the data will next talk with. The service director is responsible for allocating adequate funds for the program. The compute server’s service observer evaluates the resource status of each master PC and Fog worker node. It utilizes the catalog of warehouse services apps to determine the needs of various programs. After collecting the necessary information, the service director sets up the necessary assets in the Cloud and Fog worker nodes. The master PC protection supervisor verifies user authentication credentials obtained from a gateway device. In contrast, the Fog worker node protection supervisor monitors the Fog worker node’s smoothly protected connections with others while performing computing duties. By making a storage and resource request in the Cloud, the Cloud controller alerts the framework to instances running in the Cloud, such as containers and virtual devices. The service observer controls the flow of resources and keeps tabs on how effectively each program meets its implementation requirements in real-time. When resource utilization rises over a threshold set by the service provider or when an unforeseen problem occurs, an alert is sent to the resource management. The DL module trains a DTL-based model that processes the preprocessed data from IoT devices as the input. In addition, it considers the service director’s duties to make inferences and provide outcomes based on data accessed through gateways.

### 4.2. Framework Design and Implementation

IoT and Fog environments may be modeled with iFogSim to estimate latency, congestion, energy consumption, and cost [39]. Cost, network utilization, and perceived latency may all be tested with iFogSim for developers. FogBus bridges the gap between the Cloud, IoT, and Fog [40]. FogBus makes it possible to build IoT interfaces that are platform-independent. Administrative burdens for users, developers, and service providers are reduced by employing this. FogBus is easy to use and flexible. Pay-as-you-go Amazon Web Services (AWS) provides Cloud computing platforms and APIs for usage by individuals, groups, and government organizations [41]. The AWS data centers host Cloud infrastructure and software. Aneka develops software for the Cloud [42]. Public and private Clouds can use the .NET environment and APIs that are provided. The code developed by Aneka mimics the logic of how applications run. Models from the fields of engineering and biology are integrated into this framework. Python is an interpreted, high-level, multi-purpose, dynamic, garbage-collected language. Coding readability is enhanced by indentation. Python language may be used for both functional and object-oriented programming, as well as for more structured applications.

**Figure 4:**
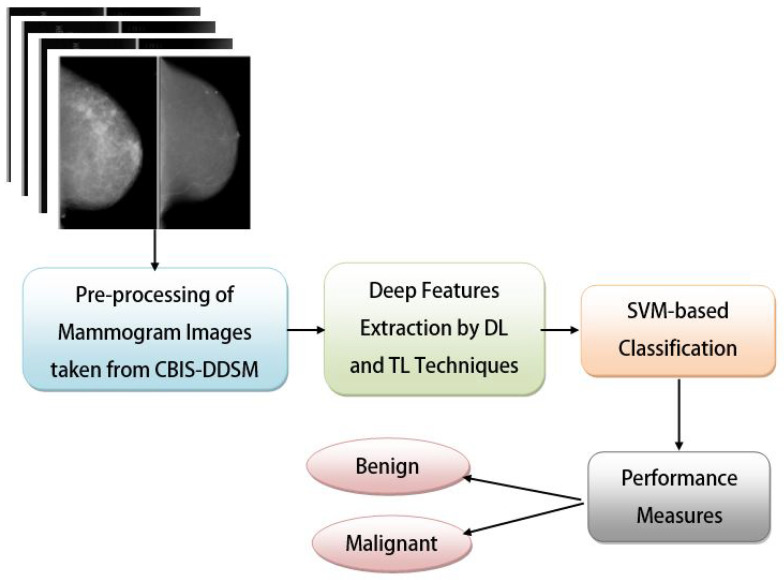
The block diagram of the proposed DTL approach.

The evaluation in this study made use of several different hardware configurations, such as the primary master PC (a Dell with a Core i3, Windows 10 64-bit OS, and 6 GB RAM), the public Cloud (AWS with a Windows server, and the Aneka platform), the gateway device (an Android v.10-powered Xiaomi A2), and the Fog worker nodes (four Raspberry Pi 4 devices, each with 4 GB of SDRAM). The proposed system was tested on a workstation equipped with Ubuntu 20.04, 32 GB RAM, a 1TB SSD, and an Intel Core i7 CPU. The proposed work’s implementation section explores many different implementations of the aforementioned elements. To train and pre-process the DTL models, Python, one of the most popular languages at present, was used. This study employed convolutional neural networks (CNNs) with ResNet50, InceptionV3, AlexNet, VGG16, and VGG19 methodologies to conduct experiments on the mammography imaging dataset retrieved from the TCIA repository. In all the cases, we used Adam as our optimizer (due to its characteristics such as adaptive learning rates, efficient memory usage, robustness to different hyperparameters, wide applicability, etc.), set our learning rate to 0.000001, set the number of epochs as 50, set batch sizes as 24, and initialized our base layers without fully connected (FC) layers. However, the depths of TL approaches were set as 50, 48, 121, 16, and 19 for ResNet50, InceptionV3, AlexNet, VGG16, and VGG19, respectively. In this study, features were extracted using one of these five TL models and then classified using an SVM. Each model employs ReLU for their input and hidden layers (as it is simple and able to alleviate the vanishing gradient problem), whereas ResNet50, InceptionV3, and AlexNet employ softmax (as it is a popular activation function and can convert the output of the last layer of the neural network into a probability distribution across different classes.) and VGG19 and VGG16 employ the sigmoid function (as it can squash the output values between 0 and 1, representing the probability of the input belonging to the positive class) in their output layers. Table 2 provides a brief overview of the DTL approach setups, and Figure 4 is a block diagram outlining the DTL approach training and measurement of performances. Five different DTL models named DTL-I, DTL-II, DTL-III, DTL-IV, and DTL-V (i.e., CNN with ResNet50 and an SVM, CNN with InceptionV3 and an SVM, CNN with AlexNet and an SVM, CNN with VGG16 and an SVM, and CNN with VGG19 and an SVM) were put to the test and their performance on the mammogram imaging dataset (CBIS-DDSM) was analyzed. Based on the results of trials measuring performance metrics, we are encouraged to use DTL-IV as the suggested DTL model in our master PC, Fog worker nodes, and Cloud data center nodes.

### 4.3. Working Principle

This recommended work is shown using several computational procedures. The master PC is the master and Fog worker nodes are the slaves in this proposed work. Devices such as the master PC, Fog worker nodes, and gateway equipment use the same network. There are three ways to communicate: using the master PC alone, using the master PC and the Fog worker nodes, or using the Cloud node only. The master PC completes the task and delivers the result in the first situation, whereas the Fog worker node performs this in the second. When the master PC and the Fog worker nodes become overloaded due to a shortage of resources, they forward to the Clouds, functioning as a gateway device. Algorithm 1 describes the main operational procedure for the proposed work. Within the predetermined framework, the hardware components of this work interact. Algorithm 2 demonstrates the internal working procedure based on the active nodes.
**Algorithm 1** Main Function of the Proposed Work**Require**: **$User_{Data}$****Ensure**: **$Binary_{Outcome}$****1**: For Active **$Gateway_{Devices}$**  **while** (1) **do**        Obtain **$User_{Data}$** using **$IoT_{EndDevices}$**        Submit **$User_{Data}$** to **$Gateway_{Devices}$**        **if $Gateway_{Devices}$** connected to **$Master_{PC}$ then**            Send **$User_{Data}$** to **$Master_{PC}$** using **$Gateway_{Devices}$**            Call Procedure ACTIVENODES( )            Obtain **$Binary_{Outcome}$**        **else**            Reset to obtain **$User_{Data}$** and submit to** $Gateway_{Devices}$** again        **end if****end while**

**Algorithm 2** Body of the Procedure Active Nodes
**Require**: **$User_{Data}$** Received via **$Master_{PC}$**
**Ensure**: **$Binary_{Outcome}$** Sent to **$Master_{PC}$**
  **procedure** activeNodes( )        Obtain **$User_{Data}$**        **if $Master_{PC}$**(Available)||(**$Fog_{WorkerNodes}$**(Available)||**$Cloud_{Nodes}$**(Available)) **then**            **if** $Binary_{Outcome}$ = = 0 **then**               Return **$Result_{Benign}$**            **else**               Return **$Result_{Malignant}$**            **end if**        **end if**        Return **$Binary_{Outcome}$** to **$Gateway_{Devices}$** using **$Master_{PC}$**.
**end procedure**



## 5. Simulation and Results

Any proposed work relies heavily on an empirical analysis of the results obtained. Defining performance standards aims to construct a class confusion matrix that compares the actual performance to the expected performance [43,44,45]. The confusion matrix is written as $TR^{P}$ and $FL^{P}$ for true and false positives and $TR^{N}$ and $FL^{N}$ for true and false negatives. Different metrics such as accuracy (*Acc*), misclassification rate (*MCR*), recall (Rec) or sensitivity (*Sen*) or true positive rate (TPR), precision (*Pre*), specificity (*Spe*) or true negative rate (TNR), f1-score (*F1S*), false discovery rate (*FDR*), negative predictive value (*NPV*), false negative rate (*FNR*), false positive rate (*FPR*), Mathew’s correlation coefficient (*MCC*), and threat score (*TSc*) were used for classification, and can be formulated as in Equations (8)–(19). An accurate prediction of the overall observation ratio is called “*Acc*”. “*MCR*” measures the proportion of incorrectly predicted observations to all observations. “*Pre*” is the proportion of accurately anticipated positive observations to all positively predicted observations. “*Sen*” is the total number of precisely detected true positives. The specific amount of genuine negatives, or “Spc”, is known. A statistical metric used to evaluate performance is called “*F1S*”, or the harmonic mean between “*Pre*” and “*Sen*”. “*FPR*” measures the proportion of false positive predictions to all negative predictions. “*FNR*” is the percentage of positive test results that result in negative results. The probability that individuals with a negative screening test do not have the disease is known as the “*NPV*”. The “*FDR*” measures how many rejected hypotheses are false positives. The measures of association for two binary variables are “*MCC*” and “*TSc*”.

(8) $$Acc=\frac{TR^{P}+TR^{N}}{TR^{P}+TR^{N}+FL^{P}+FL^{N}}$$

(9) $$MCR=\frac{FL^{P}+FL^{N}}{TR^{P}+TR^{N}+FL^{P}+FL^{N}}$$

(10) $$Pre=\frac{TR^{P}}{TR^{P}+FL^{P}}$$

(11) $$Sen=\frac{TR^{P}}{TR^{P}+FL^{N}}$$

(12) $$Spe=\frac{TR^{N}}{TR^{N}+FL^{P}}$$

(13) $$F1S=\frac{2\times Pre\times Sen}{Pre+Sen}$$

(14) $$FPR=\frac{FL^{P}}{TR^{N}+FL^{P}}$$

(15) $$FNR=\frac{FL^{N}}{TR^{P}+FL^{N}}$$

(16) $$NPV=\frac{TN^{N}}{TR^{N}+FL^{N}}$$

(17) $$FDR=\frac{FL^{P}}{TR^{P}+FL^{P}}$$

(18) $$MCC=\frac{\left(TR^{P}+TR^{N}\right)-\left(FL^{P}+FL^{N}\right)}{\sqrt{\left(TR^{P}+FL^{P}\right)\left(TR^{P}+FL^{N}\right)\left(TR^{N}+FL^{P}\right)\left(TR^{N}+FL^{N}\right)}}$$

(19) $$TSc=\frac{TR^{P}}{TR^{P}+FL^{P}+FL^{N}}$$

**Figure 5:**
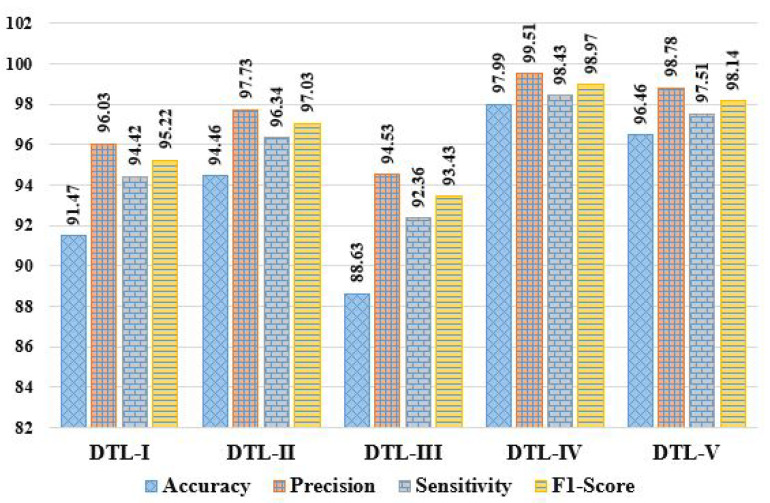
Comparative analysis of DTL approaches based on accuracy, precision, sensitivity, and f1-score.

**Figure 6:**
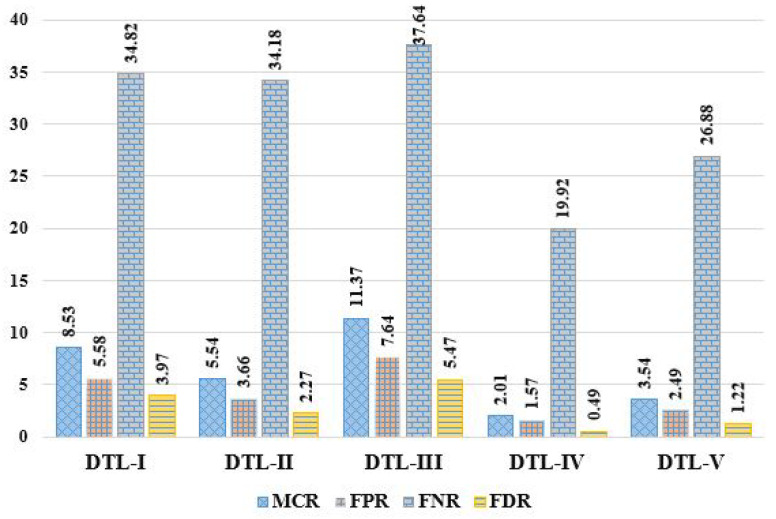
Comparative analysis of DTL approaches based on MCR, FPR, FNR, and FDR.

**Figure 7:**
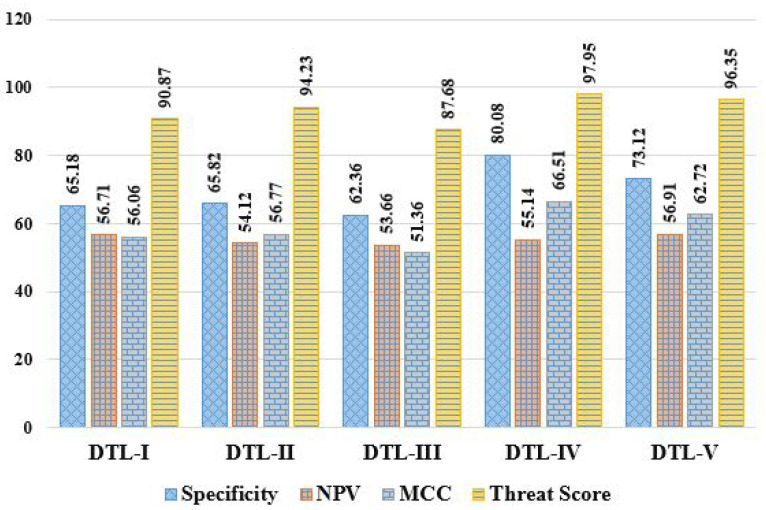
Comparative analysis of DTL approaches based on specificity, NPV, MCC, and threat score.

Here, we evaluated the mammogram imaging dataset through five DTL models named DTL-I, DTL-II, DTL-III, DTL-IV, and DTL-V, i.e., CNN with ResNet50 and an SVM, CNN with InceptionV3 and an SVM, CNN with AlexNet and an SVM, CNN with VGG16 and an SVM, and CNN with VGG19 and an SVM. The observed outcomes were then compared and contrasted, as shown in Table 3, based on some positive evaluative/performance parameters (including Acc, Pre, Sen, Spc, F1S, NPV, MCC, and TSc) and some negative evaluative/performance parameters (including MCR, FPR, FNR, and FDR). It can be observed that accuracies of 91.47%, 94.46%, 88.63%, 97.99%, and 96.46%, error rates of 8.53%, 5.54%, 11.37%, 2.01%, and 3.54%, precisions of 96.03%, 97.73%, 94.53%, 99.51%, and 98.78%, sensitivities of 94.42%, 96.34%, 92.36%, 98.43%, and 97.51%, specificities of 65.18%, 65.82%, 62.36%, 80.08%, and 73.12%, f1-scores of 95.22%, 97.03%, 93.43%, 98.97%, and 98.14%, FPRs of 5.58%, 3.66%, 7.64%, 1.57%, and 2.49%, FNRs of 34.82%, 34.18%, 37.64%, 19.92%, and 26.88%, NPVs of 56.71%, 54.12%, 53.66%, 55.14%, and 56.91%, FDRs of 3.97%, 2.27%, 5.47%, 0.49%, and 1.22%, MCCs of 56.06%, 56.77%, 51.35%, 65.51%, and 62.72%, and threat scores of 90.87%, 94.23%, 87.68%, 97.95%, and 96.35% were achieved in the case of DTL-I, DTL-II, DTL-III, DTL-IV, and DTL-V, respectively. From the experimental results obtained, it can be concluded that DTL-IV model outperforms DTL-I, DTL-II, DTL-III, and DTL-V in terms of accuracies by ∼7.13%, ∼3.74%, ∼10.56%, and ∼1.59%, respectively, precisions by ∼3.63%, ∼1.88%, ∼5.27%, and ∼0.74%, respectively, sensitivities by ∼4.25%, ∼2.17%, ∼6.57%, and ∼0.94%, respectively, specificities by ∼22.86%, ∼21.67%, ∼28.4%, and ∼9.52%, respectively, and f1-scores by ∼3.94%, ∼1.99%, ∼5.93%, and ∼0.85%, respectively. In summary, the experiments show that DTL-IV surpasses competitors based on 11 performance metrics and falls short of DTL-V in the case of NPV. The comparative analysis of various DTL approaches based on these abovementioned 12 performance parameters is depicted in Figure 5, Figure 6 and Figure 7.

Fog-enabled IoT applications’ network characteristics are strongly influenced by the computing approach or coordination level that they choose. Latency, arbitration, total processing time, jitter, network, and energy use are only some of the network metrics used to validate the proposed study and show why enabling IoT with Fog computing is crucial. In this research, we used a wide variety of configurations to assess several network metrics, including SETUP-1 with just the master PC, SETUP-2 with the master PC and a single Fog worker node, SETUP-3 with the master PC and two Fog worker nodes, SETUP-4 with the master PC and three Fog worker nodes, SETUP-5 with the master PC and four Fog worker nodes, and SETUP-6 with just the Cloud node.

**Figure 8:**
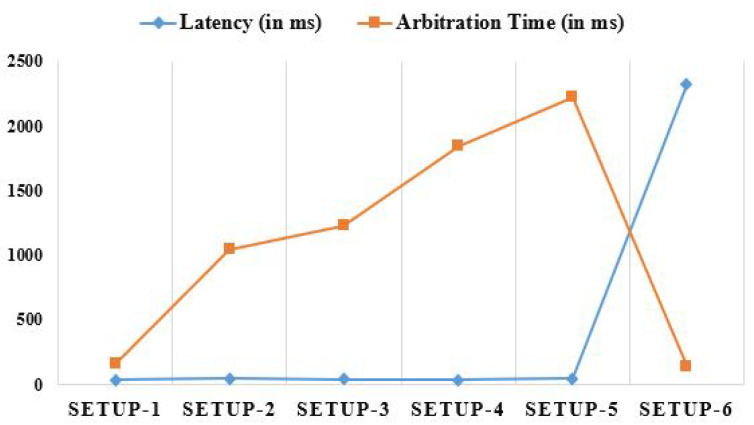
Analyzing latency and arbitration time comparatively using different set-ups.

Latency is the time it takes for data to travel across a network. Time spent in transit includes data packet collection, transmission, processing, and receipt. Combining the transmission time and the queuing delay yields the difference in latencies, as shown in Figure 8. Latency is nearly the same whether the work is sent to the master PC or the Fog worker nodes due to the utilization of solely single-hop data transfers for interactions. Since the main purpose of Fog computing is multi-hop data transport outside the network, this results in rather significant latency in a Cloud architecture. The “arbitration time” is the time it takes for the master PC to respond to gateway devices. For different thicknesses of Fog, the arbitration time is depicted in Figure 8. Directly assigning tasks to master PCs or Cloud nodes reduces the likelihood of disputes being arbitrated. Sometimes, the arbitration rate is lowered because it takes too long to disperse the load across the nodes. However, due to an increased computational capacity, Cloud processing can quickly complete tasks. Due to a decreased processing power and clock frequency, data processing on Fog worker nodes is more time-consuming.

**Figure 9:**
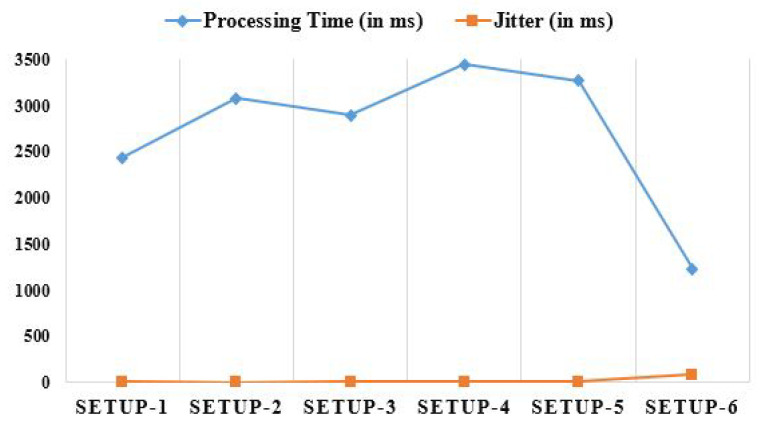
Analyzing processing time and jitter comparatively using different set-ups.

The processing time is the sum of the time it takes to initiate, complete, and return completed work to the clients. The settings are changed as well. The processing characteristics in different types of Fog are also shown in Figure 9. One obvious benefit of Cloud communications is a decreased total processing time. Jitter primarily consists of a delay over time. Jitter refers to the variation in response times experienced by individual task requests. Many real-world tasks, such as data analysis in e-Healthcare, necessitate its use. In Figure 9, we see the jitter variation across a range of values. Since the master PC controls resource management, security checking, and arbitration, the jitter is worse than when tasks are distributed to Fog worker nodes. The jitter is much larger when tasks are outsourced to the Cloud.

**Figure 10:**
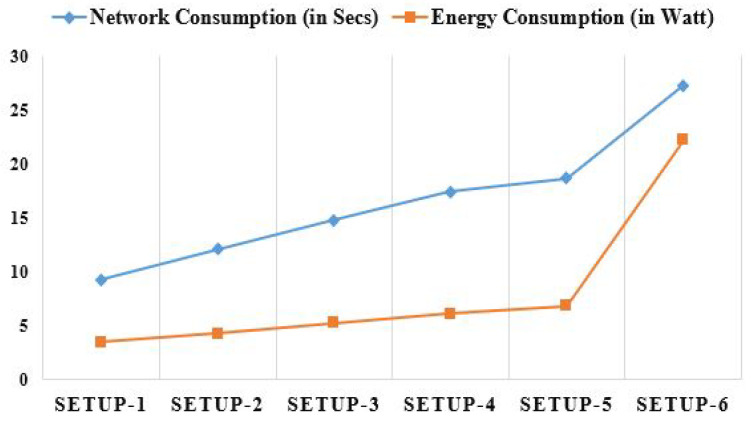
Analyzing network and energy consumption comparatively using different set-ups.

Fog computing uses fewer networks than Cloud computing. The problem affects the network utilization, including the master PC, the Fog worker nodes, or the Cloud, and the amount of Fog worker nodes. The network utilization time for the master PC and/or Fog worker nodes is much less than that of Cloud nodes because the Fog environment restricts the number of user requests transmitted to the Cloud, as illustrated in Figure 10. The term “energy consumption” is used to describe the overall system’s usage of energy. Sensors and other system components need power to function. The physical theorem may be determined by using the following formula: [46]:

(20) $$Energy_{Consumption}=Time_{TaskProcessing}\times power\left(z\right)$$

Here, $Energy_{Consumption}$ stands for energy consumed, power() stands for the function between power and the characteristic parameters for the task *z*, and $Time_{TaskProcessing}$ stands for the time it takes to complete a given task. A Cloud node requires significantly more power than a master PC or a Fog worker node, as seen in Figure 10. This is why Cloud nodes have much higher energy needs compared to Fog worker nodes. As more Fog worker nodes are added, the proposed work will require more energy. Table 4 depicts the average outcomes seen for different network parameters in different configurations based on the collected data.

The performance of the proposed framework was measured in several ways and compared to previous studies. Table 5 compares our work to numerous state-of-the-art initiatives using DL and TL approaches on mammogram imaging datasets, including details on the methodology, data types, and performance measures (accuracy, precision, sensitivity, specificity, and f1-score) utilized in the comparisons. The experimental findings demonstrate the strengths and weaknesses of the proposed method, which means that this proposed work outperforms others in some cases and falls short in other cases concerning the recorded experimental results.

## 6. Conclusions and Future Scope

The significance of Fog computing combined with IoT applications in improving people’s daily lives is quickly increasing. Considering the gravity of a breast cancer diagnosis, a patient’s ability to self-diagnose remotely via IoT applications is incredibly useful. Even though there are many issues with using Cloud infrastructures for real-time data storage, analysis, etc., formal IoT implications rely solely on them. These issues include latency, network and energy usage, etc. These issues can only be resolved by combining Fog computing with the IoT and the Cloud. This study demonstrates that a Fog-enabled system employing different DTL approaches can be used to make a timely diagnosis of breast cancer in patients. This model was trained using mammography images sourced from the TCIA data warehouse (i.e., CBIS-DDSM). Accuracy, MCR, precision, sensitivity, specificity, f1-score, FPR, FNR, NPV, FDR, MCC, threat score, latency, arbitration and processing time, jitter, network, and energy consumption are only some of the performance and network metrics employed here for experimental purposes. Integrating Cloud, Fog, and IoT computing technologies, this system enables low-latency, high-accuracy remote prediction, and the diagnosis of breast cancer. Using a large dataset of breast cancer mammograms labeled as benign and malignant, the proposed DTL approach, i.e., DTL-IV, outperformed some previous approaches based on mammogram images in terms of accuracy, precision, sensitivity, specificity, and f1-scores, recorded as 97.99%, 99.51%, 98.43%, 80.08%, and 98.97%, respectively.

This proposed research might be used to diagnose breast cancer in patients remotely. The work has a few drawbacks, such as the difficulty and high cost of implementing the proposed changes. This decentralized architecture has limitations due to relying on just one network system. Although future research on the considered dataset may explore multiclass classification, we only employed binary classification in this study. Incorporating other well-known DL and TL concepts into the suggested work may also improve it.

## Figures and Tables

**Table 1:** A short description of dataset based on binary class labels.

Class Labels					Number of Samples
	Training Samples	Test Samples	Validation Samples	Total Samples	Resolution
Malignant (Considered Binary Value: 1)	640	91	183	914	320 × 240
Benign (Considered Binary Value: 0)	609	87	174	870	320 × 240

**Table 2:** Configuration of various TL approaches taken into account in this work.

TL Approaches	Base Layer	Depth	Optimizer Used	Learning Rate	Epochs	Mini Batch Size	AF at Input Layers	AF at Hidden Layers	AF at Output Layers
ResNet50	Without FC	50	Adam	0.000001	50	24	ReLU	ReLU	Softmax
InceptionV3	Without FC	48	Adam	0.000001	50	24	ReLU	ReLU	Softmax
AlexNet	Without FC	8	Adam	0.000001	50	24	ReLU	ReLU	Softmax
VGG16	Without FC	16	Adam	0.000001	50	24	ReLU	ReLU	Sigmoid
VGG19	Without FC	19	Adam	0.000001	50	24	ReLU	ReLU	Sigmoid

**Table 3:** Observed results of various DTL models based on performance parameters.

DTL Methods	Performance Measures (in %)											
	Acc	MCR	Pre	Sen	Spc	F1S	FPR	FNR	NPV	FDR	MCC	TSc
DTL-I	91.47	8.53	96.03	94.42	65.18	95.22	5.58	34.82	56.71	3.97	56.06	90.87
DLT-II	94.46	5.54	97.73	96.34	65.82	97.03	3.66	34.18	54.12	2.27	56.77	94.23
DTL-III	88.63	11.37	94.53	92.36	62.36	93.43	7.64	37.64	53.66	5.47	51.35	87.68
DTL-IV	**97.99**	**2.01**	**99.51**	**98.43**	**80.08**	**98.97**	**1.57**	**19.92**	55.14	**0.49**	**65.51**	**97.95**
DTL-V	96.46	3.54	98.78	97.51	73.12	98.14	2.49	26.88	**56.91**	1.22	62.72	96.35

**Table 4:** Results of different network parameters as observed via different setups.

Configurations						Network Parameters
	Latency (in ms)	Arbitration Time (in ms)	Processing Time (in ms)	Jitter (in ms)	Network Utilization (in Secs)	Energy Consumption (in Watt)
SETUP-1	31.7	156.7	2435.2	6.25	9.3	3.49
SETUP-2	42.4	1046.5	3082.4	3.75	12.1	4.28
SETUP-3	36.5	1228.7	2897.5	4.50	14.8	5.26
SETUP-4	34.8	1847.5	3443.4	5.75	17.4	6.11
SETUP-5	41.3	2223.4	3273.6	8.50	18.7	6.83
SETUP-6	2318.9	142.3	1228.6	82.25	22.7	22.23

**Table 5:** A comparison of this proposed work with existing state-of-the-art works based on mammogram images.

Work	Methodologies	Dataset Used	Performance Measures (in %)				
			Acc	Pre	Sen	Spe	F1S
[15]	CNN	Breast Histopathology Images (BHIs) Dataset	88.46	85.46	95.17	82.64	79.77
[16]	Fuzzy c-means clustering algorithm	Medical CT scans	94.6	-	-	-	-
[17]	CNN	Dataset from TCIA	-	-	-	-	-
[18]	CNN	Dataset from TCIA	-	-	-	-	94.2
[19]	CNN, SM, and RF	Dataset from Kaggle	99.67	-	-	-	-
[20]	CNN and Deep CNN	Dataset from Kaggle	84	74	71	-	70
[21]	CNN and TL	Dataset from TCIA	97.0	96.0	89.0	94.0	98.0
[22]	CNN, SVM, DT, and NB	Dataset from Kaggle	98.0	-	88.5	-	-
[23]	OMLTS-DLCN	Mini-MIAS and DDSM dataset	98.50	-	98.46	99.08	98.91
[24]	CNN and TL	Breast Ultrasound Images (BUSIs) Dataset	99.10	99.10	99.06	-	99.08
[25]	SVM, KNN, RF, NB, and AlexNet	MIAS dataset	97.5	94.5	94.5	96.5	94.5
[26]	DCNN	Images from WCH, CMGH, and PHDY Hospitals	87.0	-	86.0	88.0	-
[27]	VGG-16, VGG-19, and SqueezeNet	Benchmark Breast Ultrasound Dataset	97.09	87.90	84.95	90.20	-
Proposed Work	CNN, VGG16, VGG19, ResNet50, AlexNet, and InceptionV3	DDSM from TCIA	97.97	99.51	98.43	80.08	98.97

## Data Availability

Not applicable.
